# Articular and Artificial Cartilage, Characteristics, Properties and Testing Approaches—A Review

**DOI:** 10.3390/polym13122000

**Published:** 2021-06-18

**Authors:** Mohammad Mostakhdemin, Ashveen Nand, Maziar Ramezani

**Affiliations:** 1 Department of Mechanical Engineering, Auckland University of Technology, Auckland 1142, New Zealand; 2 School of Environmental and Animal Sciences, Unitec Institute of Technology, Auckland 1025, New Zealand; anand2@unitec.ac.nz; 3 School of Healthcare and Social Practice, Unitec Institute of Technology, Auckland 1025, New Zealand

**Keywords:** articular cartilage, hydrogels, mechanical properties, tribological properties

## Abstract

The design and manufacture of artificial tissue for knee joints have been highlighted recently among researchers which necessitates an apt approach for its assessment. Even though most re-searches have focused on specific mechanical or tribological tests, other aspects have remained underexplored. In this review, elemental keys for design and testing artificial cartilage are dis-cussed and advanced methods addressed. Articular cartilage structure, its compositions in load-bearing and tribological properties of hydrogels, mechanical properties, test approaches and wear mechanisms are discussed. Bilayer hydrogels as a niche in tissue artificialization are presented, and recent gaps are assessed.

## 1. Introduction

The complex structure of healthy articular cartilage facilitates the joint withstanding the imposed pressures and retaining interstitial fluid to lessen stresses on its soft tissue while easing the locomotion and minimizing friction between cartilage mates. Avascular nature of this tissue results in unrecoverable damaged lesions and severe pain over time. Polymeric hydrogels are promising candidate materials for the replacement of the damaged cartilage. Moreover, polymeric scaffolds have been applied in interface tissue engineering and their uses have extended to bone to tendon and muscle to tendon interface reconstruction [1]. Recently, bilayer hydrogels have been developed with distinct techniques as promising artificial cartilage due to their resemblance to the native cartilage structure. Bilayer hydrogels contain bulk and lubricious layers that enhance water retention in their lubricious layer and advance tribological properties such as wear-resistance and coefficient of friction (CoF). The absence of optimum mechanical and tribological properties has been highlighted as a research gap in recent years because promoting mechanical properties results in a reduction in the tribological properties or vice versa.

In this study, summaries of recent research are covered, also essential elements in designing of artificial cartilage, common materials, required tests according to standard regulations and strengthening method are discussed broadly. Recent research has been highlighted and gaps addressed adequately. This review further discusses the fundamental resources that are considered in design of wide ranges of hydrogels specially bilayer hydrogels which have gained researchers’ attention due to their promising mechanical and tribological properties.

## 2. Synovial Joints

Synovial joints, being the most common joints in mammals, are characterized by allowing movement in multiple planes. They allow for the articulation of long bones, ends of which are covered with articular cartilage (AC), within a fluid-filled cavity. AC, incorporated with a viscous synovial fluid, is a biphasic tissue that provides extremely low friction [2]. It mitigates overstressing on the tissue’s solid phase, while dissipating energy and enabling smooth joints movements. The synovial fluid consists of hyaluronic acid (HA), glycosaminoglycans (GAGs) containing chondroitin-4-sulfate, chondroitin-6-sulfate, keratan sulfate and mobile ions and is a dialysate of blood plasma without hemoglobin [3]. The synovial fluid is contained mainly within the molecular pore spaces of the cartilage cells [4]. AC incorporates the viscous synovial fluid to mitigate shock-loadings initiated by physiological activities and body weight [5]. Hence, AC supports smooth joint movements at an extremely low coefficient of friction (CoF) [6].

## 3. The Structure of Articular Cartilage

**Figure 1:**
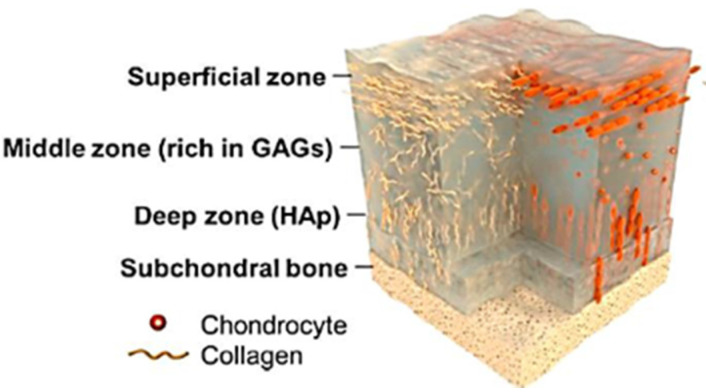
Illustration of the AC structure: superficial, transition and deep zones. Reproduced from [10].

The AC structure is complex, as the compositions of GAGs, chondrocytes and collagen are in random orientations and densities; with the main components of this composition contains water (60–85%), collagen type II (15–22%) and Proteoglycan (PG) (4–7%) [7]. Its deep zone includes hydroxyapatite (Hap) combined with collagen and chondrocyte in the vertical orientation [8], as illustrated in Figure 1. AC is a biphasic substrate categorized as a nonlinear, anisotropic, viscoelastic and inhomogeneous material [7,9].

### Zonal Categories of Articular Cartilage

AC is a soft avascular tissue with a 3–4 mm thickness and integrates three depth-dependent layers of superficial, transition and deep zones. Each layer is responsible for minimizing either the imposing load or friction of the sliding movement, as described in the following sections.

The top layer (superficial zone) contains collagen fibrils cells in the horizontal orientation, which confers high tensile stiffness and strength. This layer is just 10–20% of the tissue’s thickness [11]; both fibrils and chondrocytes are stretched along their length and surrounded at the surface with the finest size compared to the other layers’ chondrocytes [12]. This feature also custodies the tissue against high tensile stresses and prevents interstitial fluid permeation, which plays a vital role in sliding on cartilage surface mates [13]. While this layer has high water content, it has the lowest PG [14]. The superficial zone is also called the surface amorphous layer (SAL) that is acellular with no fibril content [7]. Its thickness is a few micrometers, containing proteins, glycoproteins, PGs, hyaluronic acid-protein complexes, chondroitin/keratin sulfates and lipids [15]. In summary, the superficial zone is shear resistant because of the low content of PGs and low permeability [16,17]. This layer plays a crucial role in attaining smooth sliding contact, while controlling synovial fluid diffusion rate. The transition zone is the thickest part of the tissue, contributing to 40–60% of the total thickness of the AC [18]. Collagen fibrils and chondrocytes are both ringed by an extracellular matrix (ECM) that includes GAGs [19]. Moreover, compared to the superficial zone, the transition zone has a higher PG content. The deep zone consists of orthogonally oriented collagen fibers in hydroxyapatite content and has the lowest water content. Its collagen structure is bundled together with fibers in the perpendicular direction to the articular surface. The deep zone forms an interface with the subchondral bone. The stiffness of the whole structure varies gradually through the thickness. The PG, water content and cell density are the lowest in the deep zone [18].

## 4. Osteoarthritis

Osteoarthritis (OA) is the result of AC degeneration. The recovery process of the damaged lesions is prolonged because of tissue avascularity [7]. Therefore, degenerated tissue experiences high-pressure upon sliding of bones at the joints, which results in severe pain as well as movement disorders [20]. Factors that lead to OA are aging, musculoskeletal disordering and over-pressuring due to either physiological activities or obesity [21]. It is worth mentioning that joint immobilization yields to PG loss, contributing to AC thinning [22,23].

OA is categorized into two types, namely primary and secondary. Primary OA occurs in healthy AC without any abnormality of ligaments and menisci. The reason for primary OA in the elderly is repetitive loadings on thinned AC [20]. Secondary OA, however, is due to injury, trauma or inflammatory factors [24]. In the last decade, studies showed that OA does not result only from AC disease, but also from defects in the ligament, menisci, periarticular muscles and bone [25]. AC engrossed with any of the mentioned factors instigate knee instability and alteration in joint kinematics and consequently nonuniformly distributed stresses, which initiate OA [2].

### Treatment Methods for the Cartilage Subjected to OA

The gold standard treatment for patients with OA are total knee/hip replacement (TKR/THR) or hemiarthroplasty. In hemiarthroplasty, only half of the joint in which cartilage deteriorated would be reamed, and either metallic or ceramic components are implanted. In case of hip joint damage, the acetabular cup is left intact, and damaged lesions of the femoral head cartilage would be reamed, and a metallic or ceramic cup is replaced. However, TKR or THR is not the practical solution at mid-adulthood ages due to the limitation of arthroplasty prostheses’ life span [26]. Due to the short implant service life (15–20 years), THR/TKR procedures are only suitable for elderly patients [27].

Moreover, any failure after primary surgery yields to a revision surgery. The revision surgery can be implemented for patients just once in their treatment life, since the second revision may result in the implant’s loosening [28]. Other procedures that have been developed for damaged cartilage are microfracture [29], autologous-matrix induced chondrogenesis [30], autologous chondrocyte implantation, autologous cultured chondrocytes on porcine collagen membrane (MACI) [31]. However, long-term clinical follow-ups have revealed durability issues with all the above-mentioned procedures [32]. Therapeutically, nonsteroidal drugs, corticosteroids and hyaluronic acid just relieve the pain in short-term and are pushed out of the joint within a few days [32].

Therefore, TKR/THR is the only clinical solution for older patients. However, there is not much attention for developing procedures and treatments suitable for younger patients suffering from dysfunctional cartilage, to eliminate or at least postpone the need for TKR/THR. Young patients between the age of 20–25 years old have reported the highest incidence of joint injury [3]. It turns into OA by 35–40 years old and implementing TKR/THR is high-risk at this age. If TKR, for instance, is performed at the age of 35–40 years, then based on 15–20 years’ service life of prosthesis, patients may need revision surgery at the age of 55–60 years. Revision surgery could potentially lead to disability at this age due to the loosening of the prosthesis. In this case, a novel orthopedics implant with minimally invasive surgery that could mimic the mechanical and biological behavior of the native cartilage has been highlighted among researchers as a better alternative to TKR surgery for younger patients [33].

## 5. Mechanical Characteristics of Articular Cartilage

AC can withstand imposed load under its lifetime which is estimated at 100–200 million loading cycles [34]. AC is categorized as viscoelastic due to variations of its deformation under various strain-rates [35]. It is anisotropic, since the tensile stiffness varies with the direction of loadings [36]. Furthermore, AC is inhomogeneous and performs diverged mechanical functions of tension and compression through the thickness from the superficial to the deep zone [37]. AC incorporation with the synovial fluid, which is incompressible and pressurizes noticeably, supports the significant portion of joint contact pressure [5]. These mentioned properties provide a unique cartilage structure to withstand cyclic loading from the body and transfer those loads smoothly to the bones.

AC tolerates contact pressures in the range of 3–5 MPa during the walking state in hip and knee joints [38]. Moreover, cartilage compressive and shear modulus are reported to be less than 1.5 MPa and 0.5 MPa, respectively. Its Poissons’s ratio also ranges from 0.34 to 0.48 [7,39]. AC is also classified as a poroelastic material as its stiffness is highly dependent on strain-rate [40]. Oloyede et al. [41] have reported that at low strain-rates (0.01 > ε (t)) AC response is consolidation-type deformation, which is stiffness-dependent. In contrast, at higher strain-rates (0.01 ≤ ε (t)) hyperelastic deformation mechanism is dominant that results in high stiffness according to the classical elastic deformation process [41]. Eric et al. [42] studied correlation of cartilage stiffness and strain rate and reported that strain rate increases from 2.7 × 10^−3^ s^−1^ to 3.5 × 10^−2^ s^−1^ by increasing stiffness. Their studies employing a wide range of strain rates, showed two primary mechanical responses for AC. At low strain rates, stiffness increases considerably by a minimum increase in strain rate. In contrast, at the upper strain rates regime, stiffness does not vary significantly when the strain-rate increases. Moreover, there is a critical point beyond which the stiffness does not change much by high-strain rate loading [41]. It indicates that the compressive response of AC is strain-rate dependent at low strain-rate regime.

ECM significantly affects the mechanical properties of AC. AC exhibits time-dependent responses with viscoelasticity, poroelasticity or the combination of both phenomena [43]. Research studies demonstrate that AC responds to the loads based on PGs and chondrocyte arrangement [12,44]. However, cartilage’s viscoelastic properties support the continuity of the inner tissue interactions by solid and fluid phase incorporation and fluid migration rates through the solid architecture [45]. Therefore, categorizing AC as viscoelastic or poroelastic material is highly dependent on several test factors, such as the size of the indenter, indentation depth and strain rates. Joseph et al. [43] demonstrated that the AC neither follows the classical poroelastic nor the viscoelastic model; In fact, the best model characterizing AC is a nonlinear biphasic material.

As AC is a heterogeneous, anisotropic and multiphasic biomaterial, the mechanical properties depend on its different zones. AC with three main zones and variation of collagen fibrils, PG and water contents in different layers show different responses based on the structure depth or thickness. AC with its relative strength through its thickness is in accordance with its non-homogeneity [46]. Therefore, to analyze the AC responses under loadings, the non-homogeneous poroelastic model has been recommended [47]. This model presented that the collagen fibril reinforces the cartilage through its thickness resulting in stress-strain ranges. This range is not limited just to the axial-loading direction, but also to the radial direction due to the pressurized pores by the interstitial fluid.

Hydration and dehydration are factors that affect the dissipation of pressure energy [48]. AC dissipation response is analyzed by uncoupling poroelastic and intrinsic viscoelastic mechanisms. In the dehydration state, energy dissipation reduction presents the essence of hydration in both poroelastic and viscoelastic functionality [48]. Several elements affect the mechanical properties of AC; however, researchers have circumnavigated through the complexities using customized techniques. For example, the sample-specific tissue composition has been used to predict the compressive mechanical behavior [49].

Depth-dependent mechanical properties of cartilage were also attained with optical imaging techniques such as relaxometry by MRI, which has demonstrated that under similar loading, different deformation patterns at different anatomical sites [50]. Cartilage degeneration is associated with deformation and its mechanics patterns before morphological symptoms. This finding complies with the depth-dependent mechanical properties under contact loading [50].

The cyclic loading effect on cartilage compaction was highlighted when its relaxation time was altered [40]. Moreover, static and dynamic loadings are other factors that significantly affect stress distribution over the cartilage. By dynamic loading, more uniform deformation across cartilage depth occurs, and this is because of substantial synovial fluid pressure in dynamic loading imposed on the cartilage compared to static loading. Thus, it exemplifies cartilage characteristics in reducing local strains in daily high intense physiological activities [51].

A novel method, known as Principle Component Analysis (PCA), has been developed to characterize cartilage mechanical properties with more abilities than conventional methods. This method is based on the surrounding tissue of the loaded area (L) and the transient strain (TS) of the AC during loading and unloading. L would be a benchmark to differentiate healthy and PG-depleted cartilage under loadings (deformation) and unloading (recovery) modes [52]. This framework is proving how PGs play a significant role in mechanical functioning.

## 6. Tribological Properties of Articular Cartilage

### 6.1. Wear and CoF of Articular Cartilage Components

Human knee or hip joints are subjected to up to one million cycles of loading per year during daily activities [53]. The rupture of the anterior cruciate ligament (ACL), or meniscal tears, is attributed to joints’ misalignment, consequently affecting the joint kinematics, which increases the OA risk [54]. ACL and meniscus deficiency also cause excess tribological contact stresses due to instability of the joint and immediate fibrillation on the tibial plateau [55]. Several studies have presented that cartilage properties vary as the function of local contact stresses and mechanical environment; however, tribological properties have been reported to be location-independent [56,57]. Moore et al. [58] have shown that cartilage properties are location-independent and claimed that tribological properties also vary with respect to the local mechanics of the healthy joint. They found four primary tribological responses of the healthy cartilage: first, different regions have different damage tolerances. Secondly, material properties vary remarkably due to OA diseases. Third, different properties are the results of the healthy tibial plateau and OA cartilage. Fourth, OA tissues demonstrate different tribological performances that increase the shear stresses due to mechanical failure or biomechanical degradation [58]. Since cartilage is avascular, degenerated cartilage initiated from the superficial zone and propagated to the deep zone causes destruction of the layers through the thickness, resulting in gradual material loss. Cyclic loading induces stress through the entire cartilage structure yielding microscopic damage [59]. The superficial zone in AC experiences shear stresses and cracks within its collagen fibers. Therefore, AC damage occurs when the fibers crack rate exceeds the cell repair rate [60], and this phenomenon is called AC wear-off. AC presents a rubbery surface with a meager wear rate and CoF [6] but can be escalated by the absence of lubrication, abnormal loading due to varus or valgus knee alignment, aging and excess physiological activities [61].

Wear is the amount of material loss from the surfaces due to contacting asperities and friction. In AC, the wear mechanism is categorized as adhesive, abrasive and fatigue wear [62]. Cartilage wear is because of PGs loss and alterations in the collagen network [63]. Cartilage wear could be initiated due to biochemical degradation and biomechanical factors such as knee misalignment, which induces higher pressure on either the medial or lateral side of the knee joint [64]. Most of the studies have fallen short of quantifying wear mechanism due to its complex nature; hence only frictional properties have been investigated. Several studies used metal abrader against AC to quantify wear depth, and their results demonstrated that synovial fluid incorporation with trypsin effectively protects the cartilage surface against wear [65,66]. Other studies showed that the wear rate increases with increased contact pressure, area of contact, and sliding speed [67,68]. Wear rate can be quantified by biochemical characterization of collagen and GAGs content [69]. Another method to capture wear depth and wear scar is surface topography, using scanning electron microscopy (SEM), transmission electron microscopy (TEM), atomic force microscopy (AFM), contact and non-contact profilometric methods [70,71]. Quantifying wear in AC is complex because of the deficient wear volume of soft tissues. An experiment was conducted to assess wear in AC and cartilage specimens loaded against stainless steel ball by steady sliding motion with 4.62 MPa contact pressure. Collagen loss was monitored as the wear rate indicator, and the results showed a low wear rate (0.5 µg/h at 4.62 MPa) in AC [7].

McCutchen [72] worked on the interstitial fluid and hypothesized that this fluid is the most load-bearing element in AC functioning. The author highlighted that since AC has deformable architecture, the interstitial fluid withstood most of the compressive state load. After this theory, Mow et al. [73] studied the biphasic structure and categorized it as incompressible and immiscible tissue. Katta et al. [56] then assessed that fluid could migrate through the porous AC architecture with tiny pore sizes in the range of 2.0–6.5 nm. In addition, Lai et al. [74] presented the triphasic theory, which considers monovalent ions in the interstitial fluid as the third phase. It showed three elements of fluid, solid and ion concentration are vital in identifying compressive stiffness of cartilage. Joint under compressive loading pressurize the interstitial fluid in the tissue. Such a pressure gradient in the tissue supports a significant contribution of the applied loads until the fluid is exuded away at the very beginning of the unloading period [75]. By the fluid pressurizing phase, the applied load is gradually transferred to the soft cartilage tissue, while the imposed load on the fluid is also gradually dissipated. At the equilibrium state, however, the load is tolerated by the soft cartilage tissue. Therefore, the solid phase of cartilage incorporated with interstitial fluid deprives CoF between cartilage mates. It can be maintained at a very low level as long as sufficient interstitial fluid is lubricating superficial layers of the cartilage [57].

Rehydration, contact stress, sliding contact materials and speeds are proportionally related to AC lubrication [76]. The sliding speed and stroke length are primary factors for controlling CoF and rehydration time. These factors control the wear in the cartilage surface as fluid carries the maximum load and results in a very low CoF in AC [77]. Contact stress was also reported to impact CoF significantly; increasing contact stress resulted in the reduction of CoF [78]. On the other hand, it has been shown that experimental parameters and rehydration would change the trend of decreasing CoF by increasing contact stress [57]. Consequently, Katta et al. [78] demonstrated that with increased contact stresses from 0.2 to 0.5 MPa, CoF decreased upon regular rehydration. Most of the cartilage frictional studies conducted have been based on the linear relationship between the applied load and CoF; however, further study is needed to investigate this relationship by a nonlinear trend.

Krishnan et al. [79] investigated friction in AC under cyclic compressive loading with various frequencies (0.05, 0.5 and 1 Hz). They reported that cyclic loading does not decrease CoF by increasing the interstitial fluid’s pressurization compared to the static loading. Their study showed that relocation of contact areas effectively lowered CoF rather than the cyclic loading. On the other hand, another study showed that contact stress and stroke length (for rehydration process time) affect CoF detrimentally [80].

While fluid lubrication has been highlighted as a critical element of CoF variations in experimental studies [58,81], boundary lubrication shows a remarkable improvement since cartilage is biphasic and retains fluid in its superficial layer [82]. By lubricant depletion, the CoF is mostly altered as a function of surface chemistry [56]. Boundary lubrication has been recognized for its usefulness in tissue engineering purposes, joint lubrication, cartilage substitution therapies and several other applications [75].

Biological factors also have a significant impact on CoF in cartilage. GAGs/PGs formation and existence result in fluid pressurization and consequently variation in tribological properties [83]. These materials exhibit resistance against the interstitial fluid flow, leading to a low permeability rate (~10–15 to 10–16 m^4^/Ns) [84]. Aging or joint disease leads to a reduction of GAG [85], which effectively increases the CoF rate [86]. Chondroitin sulfate is recommended in case of GAGs depletion; however, lubrication conditions must be considered [87]. Diffusing chondroitin sulfate into the cartilage reported results in a deficiency of ECM integration with chondroitin sulfate, and after imposing load, it is exuded out [56]. Collagen, another major component of cartilage, has also been reported to be effective in reducing CoF, and the lower level of collagen could exacerbate friction [88] and reduce water contents [89]. The SAL contains sulfated sugars, glycoproteins and lipids, which can be removed by wiping, resulting in higher friction than the unwiped surface [84].

### 6.2. Boundary Lubrication

Transition time in joint is shifting of dynamic to static loading or vice versa. When dynamic loading is gradually transformed to static loading, dissipating energy is mitigated by the interstitial fluid, and it permeates into the cartilage. At this stage, cartilage components absorb the synovial fluids, which initiate the boundary lubrication process [90]. Therefore, it yields to cartilage-on-cartilage contact that increases CoF.

Several studies have demonstrated the role of synovial fluid in minimizing CoF drastically under boundary lubrication regime [91,92]. Radin et al. [93] demonstrated that the proteinaceous layer has a load-bearing duty and not hyaluronic acid (HA) in the synovial fluid. In contrast, other researchers have shown that HA significantly supports the interstitial fluid in withstanding load [88,94,95]. Tests using HA on healthy and dysfunctional cartilage for both humans and bovine showed a remarkable decrease in CoF [88]. This effect is limited to lowering CoF in dynamic loading, even under static pressure, while boundary lubrication occurs. HA penetrates into the cartilage structure and surrounds the chondrocytes, which preserves the CoF levels [95].

Lubricin, a mucinous glycoprotein, is another component of synovial fluid has been reported that lack of lubricin in synovial fluid resulted in inadequate boundary lubrication and increases wear in cartilage [70]. This research showed that in the presence of lubricin, adhesion between contacting cartilage is minimized, and this process yields to decreased friction upon boundary lubrication [70].

As another component of synovial fluid, phospholipids contributed significantly to boundary lubrication due to the hydrophobic nature of its fatty acid [96]. Hills and Crawford [97] reported that phospholipids are a component of lubricin in the boundary lubrication, whereas lubricin and HA only supported the phospholipids. Furthermore, Pickard et al. [98] demonstrated that elimination of phospholipid from the cartilage increases the CoF of cartilage minimally. Their study was just limited to the short time; however, no remarkable effect was reported at a prolonged time regarding the cartilage friction properties.

According to the literature, all mentioned components of synovial fluid effects boundary lubrication, and isolating any component can compromise the boundary lubrication process. Moreover, the biomechanical and biochemical synergies may also be insufficiently controlled, as it is in a synovial joint. Nevertheless, all these findings are the expedient benchmark to characterize wear and CoF in AC.

## 7. Tissue Engineering of Articular Cartilage

Cartilage tissue engineering has been investigated extensively by researchers since this tissue is avascular, and confined migration of chondrocyte reduces its self-recovery considerably. Therefore, the essence of artificial cartilage motivates researchers to design and manufacture materials mimicking mechanical and tribological responses of the native cartilage. Polymeric hydrogels have been highlighted as candidates for this application as they resemble the biomechanical, biochemical and architectural properties of native cartilage [99]. Hydrogels have also appealed to researchers due to their biocompatibility [100], nontoxicity effects and no stimuli on the immune system [101]. Hydrogels are categorized as natural and synthetic and can be modulated with cell-free or cell-laden scaffolds. Some of the cell-free scaffolds have been presented with the use of bacterial nano-cellulose [102], polyethylene glycol (PEG) in combination with HA [103], collagen-hydroxyapatite hybrids [104], aragonite-hyaluronate membranes [105], acrylamide (AAm) hydrogels [106], alginate (Alg)/chitosan compounds, agarose/polyglycolic acids (PGA) [107], and porous polycaprolactone (PCL) [108]. The mentioned scaffolds were used clinically; however, after clinical follow-up in the longer term, they were rejected due to the lack of strength and durability. The following sections describe some of the common materials used in the manufacture of hydrogels.

### 7.1. Hydrogel Materials

#### 7.1.1. Hydrogel Classifications

Hydrogels are classified based on raw materials, chemical composition, physical structure, type of crosslinking, physical appearances and electrical charge, presented in Table 1.

#### 7.1.2. Polymer Materials Used for Articular Cartilage Synthesis

Table 2 presents comparative advantages and applications of wide ranges of materials in synthesizing polymeric hydrogels for articular applications.

### 7.2. Synthesis of Hydrogels

#### 7.2.1. Crosslinking Hydrogels

Various crosslinking approaches have been reported to synthesize hydrogel, such as chemically modified process, crystallization process, free-radical polymerization and ionic polymerization [127,128]. Table 3 presents four prevalent approaches that are used to synthesize hydrogels for medical applications.

The major limitations for the biomedical application of hydrogels are the non-biocompatibility of some hydrogels and potential toxicity of residual unreacted small cross-linkers in chemically crosslinked hydrogels [135]. However, among methods mentioned above, free-radical polymerization is a prevalent method used to synthesize hydrogels for biomedical applications [136].

#### 7.2.2. Free Radical Polymerization

Free radical polymerization (FRP) is a capable technique to produce about 50% of monomers to polymers [137]. The major advantage of FRP is its insensitivity to monomer and impurities compared to ionic polymerization [138]. It can be applied in normal room conditions, which minimize the cost of production. A broad range of monomers can be utilized in FRP to turn to polymers which is the great advantage of this technique [139].

Free radical polymerization involves the conversion of monomers into polymers through the initiation, propagation and termination steps. The ‘’initiation’’ process involves the production of radicals that start the reaction with monomer. An existing free-radical interacts with the monomer resulting in a new radical, which in turn opens another molecule monomer. This process repeats to result in a polymer, and this step is called ‘’propagation’’. The polymerization reaction stops when the last radical of one polymer chain meets another chain with the free radical, and when they combine, the polymerization process is completed, hence the “termination” step [140].

### 7.3. Bilayer Hydrogels

**Figure 2:**
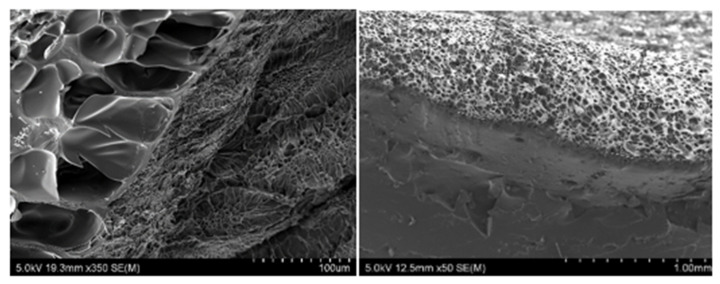
PAAm-Alg bilayer hydrogels: cross-section view.

Bilayer hydrogel consists of a porous architecture layer integrated with a bulk layer covalently. The porous architecture is the result of the interruption in the polymerization process. The porous layer benefits hydrogel in water retention, impact on diffusion rate, minimizing CoF and wear rate [104,119]. Gong et al. [141] developed a bilayer hydrogel with varying crosslinking degrees in the top layer. A lower degree of crosslinking resulted in high porosity and the hydrogel had a higher fluid retention capacity, which consequently minimized the CoF. The bilayer architecture formation in hydrogels is due to branch dangling chemical phenomenon [141]. A branched dangling polymer chain is achieved by polymerizing the monomers, while in contact with a hydrophobic surface. Hydrogen-rich moieties are located within close vicinity of the hydrophobic surfaces yielding a low density highly porous structure. This is attributed to the high concentration of hydrogen affecting the propagation step of polymerization. The bulk area, which is far from the hydrophobic surface, could accomplish the polymerization process due to hydrogen deficiency in this zone. Consequently, a very dense structure is formed, and the bulk area’s strength enhances compared to its porous counterpart [142,143]. The SEM image of a bilayer hydrogel cross-section is presented in Figure 2.

### 7.4. Mechanical Testing of Articular Cartilage and Hydrogels

AC as a soft tissue articulates the full range of motions and experiences complex loading scenario, which is compression, tension, shear and friction [144]. Most studies focused on assessing recovered tissue based on biochemical, gene expression, or histological aspects [145]. Comprehensive protocols for mechanical evaluations showed a lack of standardization for their unit reference. Therefore, remarkable tolerances in the reported data are inevitable. The compression testing is categorized as unconfined, confined and in situ. For the confined compression test, a porous plate or indenter is used to let fluid flow out of the tissue.

Four test configurations are commonly used to characterize cartilage mechanical responses: ramp, stress relaxation, creep and indentation tests. Jay et al. [70] reported that the most utilized test configuration in studies from 2009 to 2018 were: ramp, stress relaxation and creep. Thus, the ramp test has been configured to simulate the load-bearing properties of the tissue. After recording the stress-strain response by the ramp test, the first-order differential equation of the curve, which is the slope of the stress-strain curve, results in the tangent modulus of the tissue. Tangent modulus quantifies softening and hardening of the material and plastic deformation beyond yield stress [132]. Softened materials endure a higher load before ultimate failure compared to hardened materials and are suitable for replacing tissues that undergo large deformations [146]. Two factors that considerably affect the tangent modulus are strain rate and strain point. Healthy knee cartilage typically experiences average strains under 10% [36] and a maximum of 17% [147]. The tangent modulus is estimated by laying on the curve less than 10% strain at different strain points, hence, tangent modulus data would not be clinically helpful. However, it shows at each strain point how hard or soft tissue responses are. This is relative to the micro-architecture of the tissue matrix, porosity and fluid flow rate within the matrix [148].

It has been highlighted that compression tests are essential with modeling of viscoelasticity responses according to required tests of the United States Food and Drug Administration (FDA) and International Cartilage Repair Society (ICRS) [149,150]. Moreover, the American Society for Testing and Materials (ASTM) standard is focused on confined creep testing as a requirement for mechanical evaluation of designed tissues [151]. Alternatively, creep or stress relaxation is needed to quantify material properties recommended by ASTM. A systematic review of literature from 2009 to 2018 [152] showed only 11.4% of studies had performed stress relaxation or creep tests, which demonstrates that most studies did not meet the requirements of the FDA and ICRS guidance documents.

### 7.5. Tribological Testing of Articular Cartilage and Hydrogels

In the tribology testing of both native and engineered cartilage, there are two methods of testing the lubrication properties; the first method is sliding mate with a specified stroke length, which yields to matrix deformation. The CoF would be very low as the fluid resistance is against imposed load in the active deformation region. It is reported that the load support can be analyzed by Peclet number, where low friction occurs by the condition of Pe > > 1 and connective fluid velocity surpasses diffusive fluid velocity [5,75]. The second method of lubrication analysis is aimed more at boundary lubrication which is a stationary contact area. In this method, a sample is compressed to a solid mate, and CoF is recorded as the fluid pressure drops to the ambient pressure [81]. Therefore, interstitial fluid pressure lessens, and only contact pressure between two solid mates determines the CoF associated with the biochemical and articular surface. Thus, this method is suitable to analyze boundary lubrication and its biomolecular interactions. It is worth mentioning that a correct interpretation of using the two methods is necessary and depends on the surface and pressuring mechanism. If a tissue provides excellent permeability, which increases the localization of lubricants, it will have a relatively low CoF in stationary and high CoF in migrating contact areas. In contrast, a tissue with a remarkable pressurizing fluid mechanism but poor in boundary lubricants would have a relatively low CoF in migrating contact area and high CoF in stationary contact area [152].

## 8. Mechanical Properties of Hydrogels

Crosslinking process within polymer chains improves the compressive strength, stretch-ability and toughness of the hydrogels to withstand shear or compressive stresses [141]. There are two conventional crosslinking approaches. Covalent crosslinking enhances materials’ strength and dissipates mechanical energy against deformation, whereas ionically crosslinked augments self-healing properties and controls degradation of the polymeric network [26]. Furthermore, it was reported that ionically crosslinked hydrogels using Fe^3+^ or Al^3+^ also exhibited enhanced mechanical strength [153]. Crosslinking density affects the polymer chain length, and consequently, different properties can be achieved [154]. The dangling chains phenomenon exploits the hydrophobicity and hydrophilicity interaction to form a low-crosslinked density that improves lubricious fluid retention. The high-crosslinked density, however, results in a bulk layer that enhances structure load-bearing [153]. Furthermore, interpenetrating polymer networks (IPNs) are formed by interpenetrating entanglement of two or more crosslinked polymers. A semi-IPN results when only one polymer in the system is crosslinked, whereas, crosslinking of all polymers in the system results in full-IPN. Hence, the mechanical strengths of hydrogels in the form of full-IPN structure is superior compared to semi-IPN structures [155].

An improvement in the mechanical properties mitigates the lubrication properties of hydrogels. Subsequently, research on having a load-bearing structure with a sufficient lubricational threshold has not yielded the desired success; therefore, this subject warrants further research attention. It has been proven that monomers molar ratio, initiator and crosslinking degree determine the mechanical properties of hydrogels [156]. Zhang et al. [119] reported that the mechanical properties of bilayer hydrogels improved notably by meticulously increasing monomer (acrylic acid, AAc) content. Increasing the amount of AAc, resulted in ultimate tensile strength and elastic modulus increase. However, when AAc was more than 50%, hydrogels become very brittle and stiff, resulting in inferior tensile properties [132], and were not suitable for practical applications. Xu et al. [157] found that the titanium nanocomposite hydrogels having 10% AAc had significant tensile strength and enhanced water stability (low swelling ratio) compared to the higher molar percentage of AAc. Optimum AAc amount improves the mechanical strength and affects the nonlinearity of the hydrogels, which is a premium consideration in tissue engineering applications [158]. Arjmandi et al. [26] reported that their hydrogel’s mechanical properties improved by increasing crosslinking concentration up to 21% and 32% for elastic modulus and hardness, respectively. Trivalent cations (Al^3+^ or Fe^3+^) also presented a momentous factor in increasing the strength and stiffness when hydrogels were synthesized using alginate monomer [159].

Among polymers, alginate and polyacrylamide (Alg/PAAm) have been reported to provide a high level of toughness and stretch ratio [160]. The elastic properties, furthermore, were reported to be similar to that of AC. Alg/PAAm also proved a 3-fold decrease in CoF compared to either Alg or PAAm as single network hydrogels [161]. Alg, however, has some disadvantages such as low tensile properties and difficulty in sterilization and controlling the hygiene process during synthesis. Its impurities may also affect material properties [162]. To sum up, optimum amounts of AAc, AAm, Alg and relevant crosslinking ratios would significantly improve both the mechanical and tribological properties of hydrogels.

### Viscoelastic and Poroelastic Relaxation

Viscoelastic and poroelastic are associated with the rate of fluid migration within the networks, and their interaction with polymer chains results in dissipating energy [163]. Therefore, the assessment of hydrogel materials and their viscoelastic or poroelastic relaxation response is essential in designing tissues where they are subjected to high-impact loads. Hydrogels are formed by fiber networks similar to fibrin and collagen in AC and can be categorized as viscoelastic due to the exhibition of stress relaxation [164]. A nano-porous hydrogel structure, such as acrylamide hydrogels, performs minor viscoelasticity and is nearly elastic [46]. It was reported that stress relaxes promptly when the hydrogel is crosslinked ionically compared with covalently crosslinked [165]. More details were reported by Zhao et al. [165] and showed that binding and unbinding of alginate hydrogels that are crosslinked ionically show quicker stress relaxation compared to the covalently crosslinked. By exerting a force that results in unbinding of ionically crosslinked fibers, divalent cations detach from the anions of alginate chains and re-bond with another anion. In contrast, the covalently crosslinked network does not detach and re-attach fibers. Thus, instead of detaching, it yields to a longer time to relax the stress [166]. The covalently crosslinked hydrogels exhibited time-dependent mechanical properties.

It is highlighted that an abundant amount of water in hydrogels also affects viscoelastic responses. Fluid motion within the network would significantly impact dissipating energy from external loadings [165]. Hong et al. [167] formulated a coupled mass transport theory and large deformation within the hydrogel network. The motion of fluid inside the network and the resistance of the porous structure against the fluid migration yield to macroscopic mechanical relaxation, which is different from relaxation resulting from structural deformation in the network. This phenomenon is called poroelasticity and is characterized by diffusion coefficient D of the fluid in the network [168] and can be obtained by the following equation:

D~Er2/η(1)

where E is the elastic modulus, r is the pore radius of the polymer network and η is the fluid viscosity in the hydrogel. According to the equation, the rate of relaxation depends on poroelasticity. As mentioned above, regarding the fluid migration, the smaller pore size results in slower fluid migration and thus slower stress relaxation. Therefore, diffusion rate D, and geometric scale L of the sample are inversely proportional to the time of stress relaxation. A smaller L yields to a faster stress relaxation due to the fluid migration at a shorter distance. However, the rate of deformations of a hydrogel is independent of the geometric scale [165]. In addition, viscoelastic responses are always attributed to fluid flow and network deformation. Therefore, when L >> √(Dτ_v) which was obtained for hydrogels when the sample scale is large enough to prevent the fluid from migrating to the end, viscoelastic relaxation occurs before poroelastic relaxation [169]. If we consider two states of time required for hydrogel to reach viscoelastic and poroelastic relaxation, therefore, t~τ_v is the time of viscoelastic relaxation from deformation and t~τ_p is the time of poroelastic relaxation resulting from fluid flow. τ_v is the time of viscoelastic relaxation and τ_p is the time of poroelastic relaxation. Therefore, it is essential in the design of artificial cartilage to assess the viscoelastic and poroelastic time of relaxation based on material properties.

## 9. Tribological Properties of Hydrogels

Wear is the loss of material, a continuous damage process due to the sliding of contact mates throughout cycles. Wear, V, is defined as the total volume of material loss. Wear rate (w) reported by Archard et al. [170] is defined as volume loss per unit sliding distance. Archard’s equation predicts that the wear rate is proportional to the normal contact pressure and inversely proportional to the hardness of the material surface:

w = V/s = K P/H(2)

where V is the total volume loss in [mm^3^], P is the normal load in [N], H is the hardness of the material, s is the sliding distance and K is the so-called wear coefficient, a constant that is usually determined by experiment for two specific contact partners under certain environmental conditions.

A conventional system for analyzing tribological parameters is the pin-on-disk tribometer, where a small pin slides on a larger circular disk. The sliding motion is between the specimen and the rotating disk. Several types of motions and sliding between solids have been introduced (i.e., sliding wear, rolling wear, impact wear and oscillation wear) [171]. The dominant wear mechanisms are abrasion, adhesion, surface fatigue and tribochemical reactions. Abrasive wear is the subtraction of a soft material by a hard adjacent surface [172]. The most substantial part of the abrasive wear is caused by tangential sliding motions and removal of the microscopic asperities. Adhesive wear is associated with an increase in the CoF, µ between the interfaces [172]. Up to µ = 1.0, the presence of friction can be explained by adhesion itself, which means that frictional resistance is caused by asperities coming into contact and adhering to one another. Corrosive wear is a mechanism of materials and environment interface; development of worn surface may yield to different scenarios as relative motions of the bodies. Finally, wear due to fracture is a description of removal of chunks of material due to microcrack occurs within material either due to surface cracks or subsurface cracks [57].

Bilayer hydrogels that consist of a bulk layer for bearing load, and a thin porous layer to retain fluid and minimize the CoF have been developed recently [119,153]. In these bilayer hydrogels, the bulk layer exhibited significant compressive strength up to 0.35 MPa. The reciprocating sliding test reported a 0.038 CoF associated with its lubricious layer. However, the lubricious layer was worn after a few thousand cycles due to its low network density. Surface network density is inversely proportional to water retention, which in turn influences the CoF reported by Zhang et al. [119]. Crosslinking density is proportionally related to the mesh size and showed a remarkable correlation at the transition of low to high frictions [173].

In an earlier study, the lateral and normal friction forces were not directly correlated to the stiffness but varied with the hydrogel architecture and composition [174]. The contact pressure and pore pressurization within interconnected channels are the key factors that control hydration levels in tribological assessments [168]. The contact pressure experienced by AC was reported in the range of 0.1–2.0 MPa in the hip and knee joints [76,175]. By increasing contact stress on AC, the CoF decreases [78]. However, research showed that experimental parameters and rehydration would change the trend of decreasing CoF by increasing contact stress [176].

Beyond CoF values, the determination of lubrication mechanisms in hydrogel has rarely been addressed. The effects of load and speed on lubrication regimes have been studied with the aid of the classical engineering Stribeck curve [177]. They found that hydrogels are not covered the engineering Stribeck curve regimes, and the main regimes were developed: mesh-confined, elastoviscous transition and fluid film.

In the engineering system, a prompt transition occurs over narrow ranges of the Hersey number, which is also the dimensionless fluid thickness. It is worth mentioning that stiff engineering materials have elastic moduli in the scale of GPa; therefore, the hydrodynamic fluid film would form by increasing speed or decreasing load. However, hydrogels with conformational surfaces with respect to contacting mate and a much lower range of elastic modulus (kPa) do not fall into this lubrication regime. Therefore, hydrogels are viscoelastic materials, and their wear behavior is similar to that of rubbers; thus, fatigue and adhesion wear mechanisms are dominant [178].

Furthermore, effects of applied load and sliding speed on shifting wear mechanisms have been investigated recently, and it was shown that unlike applied load, sliding speed has a minor influence on the wear mechanism [179]. Addressing these tribological properties is essential to ensure hydrogels under various contact pressures and sliding speeds can perform similar to AC [177].

A knee joint represents a situation of soft elastohydrodynamic lubrication (EHL). Artificial implants are examples of hard EHL. Hard EHL can be very successful in tribological situations, but only when the lubricating fluid has superior high-pressure rheology. This is not the case for synovial fluid, [180]; thus, the soft EHL results in thicker lubricant films than hard EHL in vivo. To this end, a porous architecture of polymer would mimic natural cartilage in terms of EHL lubrication and yields significant performance to conventional fully dense polymers. The CoF associated with different polymeric materials are presented in Table 4.

## 10. Strengthening of Hydrogels with Nanoparticles

TiO_2_ nanoparticles (TiO_2_ NPs), due to their low toxicity, excellent biocompatibility, low cost and high-level stability, have been explored for the synthesis of polymeric hydrogels for medical applications [185]. However, due to the hydrophobic nature of these nanoparticles, having a homogenous solution that affects mechanical and tribological properties would be challenging. These challenges are because of TiO_2_ NPs surface and electrostatic attraction among particle molecules [186]. TiO_2_ nanoparticles tend to agglomeration or aggregation due to solution ionic strength (IS), pH level, surface charge or coating [187]. Using different techniques may affect the tendency of nanoparticles to aggregation. Some researchers have reported these techniques, which are ultrasonic irradiation, stabilize TiO_2_ NPs in an aqueous medium, electrostatic stabilization, controlling pH level of the solution by neutralizing acidity level, and coating the surface of nanoparticles by surfactants [188]. Moreover, overcoming the van der Waals attraction of nanoparticles by utilizing steric or electrostatic stabilization is the critical factor to suppress nanoparticle aggregation or agglomeration effectively.

Ultrasonic irradiation was an effective method to disperse NPs, which depends on solvent type, concentration and suspension volume. Two ultrasonic irradiation methods, bath and probe sonications, are commonly used, although probe sonication showed a better result [187]. Even using probe sonication is not the permanent solution to suppress aggregation. Stabilizers were reported to have prolonged effects on dispersed particles [189]. As mentioned earlier, steric and electrostatic stabilization takes place when charges accumulate by the particle surfaces. More than 30 mV or less than −30 mV surface charge on the TiO_2_ NPs yields no aggregation. Moreover, having higher than 1% TiO_2_ NPs concentration in the AAm-based hydrogels composition resulted in sedimented particles even if a long homogenization process was used [190].

Some monomers of hydrogel compositions have a high acidity level, for instance, AAc, which can affect NPs dispersion. The hydrodynamic size of nanoparticles can be tuned by modifying the pH level of the solution. TiO_2_ and SiO_2_ particles have a positive surface charge when the pH level is low, and on the opposite, negative surface charge when the pH level is high [191].

### TiO_2_ and Silica NPs Mechanical and Tribological Properties

Chemically crosslinked co-polymeric hydrogels have been reported to have superior mechanical properties compared to the conventional chemically crosslinked homo-polymeric hydrogels. This has been attributed to the formation of more uniform and compact networks in the co-polymeric hydrogels [154]. However, nanocomposite hydrogels, loaded with nano particles (NCHs), reported higher strength, improved sliding wear resistance, anisotropy and potential self-healing property compared with double-network hydrogels (DNHs), topology hydrogels (TPHs) and micromolecular microsphere hydrogels (MMHs). The swelling ratio is a crucial factor for hydrogels in biomedical applications, which supports water-stability within the hydrogel and can be achieved by utilizing titania NPs [154]. The superior mechanical strength of hydrogels is associated with the equilibrium swelling state. Seddiki et al. [133] reported that TiO_2_ NPs and a high dosage of crosslinking agents (15%) are vital factors affecting swelling ratio. Furthermore, it has been reported that carboxyl groups formed complexes with TiO_2_ NPs via different methods to crosslink polymer chains [192].

The concentration of TiO_2_ NPs is a critical point in the reinforcement process since this substrate act as a crosslinker. The higher concentration of NPs, which is inversely proportional to the structure mesh size, would produce a higher degree of crosslinking [190]. Consequently, with smaller mesh-size, hydrogels would imbibe less fluid in the networks, which affects stress distribution over the structure. Due to this fact, the poroelasticity and viscoelasticity relaxation time would also be affected.

Silica nanoparticles (SNPs) have also been utilized to synthesize artificial cartilage and have demonstrated appreciable mechanical and biological properties [143,193]. Incorporating SNPs within polymer networks improves tissue adhesion, stiffness and shear modulus [194]. Furthermore, SNPs, interlaced with polymer chains, enhances hydrogel elasticity [195]. Zareie et al. [196] showed that by increasing SNPs amounts in the polyacrylamide networks, the number of tie points in each entanglement increased, and the compressive strength of hydrogel reached 26.2 kPa.

In addition to improving mechanical strength, SNPs have promoted the degree of crosslinking in very weak chemically crosslinked PAAm hydrogels, which have interestingly presented the ability of SNPs to function as a crosslinker [195]. Arjmandi and Ramezani [146] reported that SNPs interact with PAAm chains resulting in network crosslinks through hydrogen bonds.

Unlike other NPs, SNPs showed a significant impact on initial shear modulus and viscoelastic properties since they could immobilize the polymer chains and form NPs-polymer interphases [197]. SNPs reported increasing the number of tie points in each entanglement, which results in the improvement of the compressive strength [196]. SNPs also enhance slower chain kinetics and relaxation due to tough NPs-polymer bonds [146]. Polymer bonds relax promptly when NPs are located far from chains [198]. Viscoelasticity of the SNP loaded nanocomposite hydrogels (NCHs) was studied extensively and found to be similar to that of AC [195]. AC exhibits a time-dependent response associated with viscoelasticity, poroelasticity or the combination of both phenomena [43,199].

Tribologically, SNPs showed the dominance of adhesion mechanisms rather than other wear mechanisms, although fatigue wear took place with surface pitting at higher applied loads [200]. Utilizing 1–4% SNPs into the PAAm-alginate network resulted in low Cof values in the range 0.0035–0.0055, which is comparable to the CoF of AC (0.0001) [146]. It is attributed to the strong interfacial NPs-polymer bonding in the hydrogel matrix. The contact pressure and pore pressurization within interconnected channels are the key factors that control hydration levels in tribological assessments [168]. SNPs also affect mesh patterns, and therefore, are strongly correlated with the lubrication regimes [200].

## 11. Conclusions

In this paper, a comprehensive review of the literature for the AC is presented. First, the architecture of the AC, its compositions and the role of each component on mechanical and tribological properties were discussed extensively. It was explained that damaged cartilage cannot recover itself due to its avascular nature. Then, osteoarthritis roots and treatment methods were presented with conventional TKR/THR solutions as the ultimate treatment being highly invasive and with significant disadvantages especially for younger patients, and the need for revision surgery due to the limited service life of TKR/THR implants were discussed. To address the gap in treatment of younger patients with OA, developments of artificial cartilage by different synthesizing processes, materials and their pros and cons were described. The required standard and necessary tests for artificial cartilage to assess its mechanical and tribological properties based on the International Cartilage Repair Society (ICRS), Food and Drug Administration (FDA) and American Society for Testing and Materials (ASTM) were briefly reviewed. Viscoelastic properties were found as the critical point in the design of engineered soft tissues and the techniques to tune viscoelasticity to perform optimum responses under different loading scenarios were reviewed. Advanced bilayer hydrogels were discussed as a promising candidate for artificial cartilage. Both the load-bearing and lubricious layer were investigated recently; however, the weak point of the proposed lubricious layer was found to be its limited strength and service life under cyclic sliding tests.

Polymeric hydrogels have indeed provided a practical alternative to AC when OA treatment is considered. This is largely attributed to the progress attained in finding the appropriate combinations of materials as well as techniques for the synthesis of hydrogels with mechanical and biochemical properties of natural cartilage. Polymeric hydrogels stand to emerge as an attractive technology for AC replacement applications. Even though highly promising, the application of hydrogels in AC replacement are not free from challenges of biocompatibility. It is, therefore, imperative that attention be diverted to understanding the degradability of synthetic polymeric networks and the interaction of the hydrogels with cells in physiological conditions. A further area of innovation would be addressing the fabrication challenges of hydrogels which will make them safer and ready for clinical use. The mechanical properties of hydrogels are an important consideration for AC replacement application; however, mechanical characterization of hydrogels have been limited to mainly cell free scaffolds. Cells’ encapsulation can potentially dictate profound changes in mechanical properties of hydrogels. Hence, mechanical characterization of cell seeded hydrogels should be a consideration for the future.

## Figures and Tables

**Table 1:** Classification of hydrogels.

Classification of Hydrogels Based on	Ref.	Subdomains	Features
Source		Natural origin Synthetic origin	—
Polymeric composition	[32,109]	Homopolymeric hydrogels	Network formation by single species of monomer.
		Copolymeric hydrogels	Network formation by various monomer species with at least one hydrophilic monomer.
		Multipolymer hydrogels	Synthesized by two independent crosslinked natural or synthetic polymer.
Physical structure and chemical composition	[110]	Amorphous	- Non crystallized polymer chains contain an abundant amount of water. - Mechanically weak. - Very soft and homogenously heparinized.
	[111]	Semi-crystalline	- Moderately water-swollen hydrogels. - Mechanically stable and performing melt-processability, and self-healing function.
	[112]	Crystalline	- Structurally unique and hierarchical. - Morphologies depend on their molecular architectures.
Type of crosslinking	[113]	Chemically crosslinked (permanent joints)	Covalent bonding between polymer chains.
		Physical crosslinked (transient junctions)	Physical interactions between chains result in chain entanglement, hydrogen bonding, hydrophobic interactions and crystallite formation.
Physical appearances post-polymerization		Matrix, film and Microsphere	—
Network electrical charge	[114]	Non-ionic (neutral)	Less toxic to the cells in vitro.
	[115]	Ionic (including anionic or cationic)	High strain sensitivity and many superior mechanical properties.
		Amphoteric electrolyte	—
	[116]	Zwitterionic (polybetaines)	Anti-polyelectrolyte” behavior, unusual pH sensitivity and temperature sensitivity.

**Table 2:** Commonly used polymers in articular cartilage synthesis.

Polymers	Ref.	Advantages	Applications
Acrylamide	[117]	- High level of toughness and stretch ratio - Similar elastic properties to that of native cartilage	The base of the most polymeric hydrogels.
Acrylic Acid	[118]	- Great impact on tensile strength and elastic modulus - Usage amounts effects on more crosslinking and shorter polymer chains, yields higher toughness - Usage results in nonlinearity in mechanical response - High capacity in water retention for swelling applications	Used in synthesizing hydrogels.
METAC *	[119]	- Deprive wear loss rate - Retain water in the hydrogel matrix and decrease CoF	Utilized in hydrogels that must be riched of water in prolonged time in biomedical and pharmaceutical applications.
Hyaluronic acid	[120]	- Tissue healing, expansion of cell proliferation and migration - Angiogenesis - Inflammatory response control	For treatment purpose of osteochondral diffusion, enhancing chondrogenesis within the damaged tissues.
Cellulose	[121]	- Special fibrous nanostructure, with excellent mechanical and physical characteristics	Methylcellulose includes producing thermosensitive hydrogels applicable in drug delivery systems.
Dextran	[122]	- Biodegradable - Biocompatible - Bioadhesive	Wound healing, Relief patient pain, Hard for installation and removal.
Alginate	[123]	- Biocompatible - Availability and reproducibility - Low cost	Wound healing, Encapsulation of therapeutic agents, Tissue engineering applications.
Chitosan	[124]	- Biocompatibility - Biodegradability - Non-toxicity - Biological characteristics	Hydrogel synthesized by Chitosan and beads applicable to embedding drugs for transport bioactive substances. Drug delivery applications.
Gelatin	[125]	- Biopolymer’s biotoxicity - Biodegradability - Potential to induce cell migration	The optimal candidate for applications for extracellular matrix (ECM), 3D structure, Cell transplantation.
Polyvinyl alcohol (PVA)	[126]	Biocompatibility Biodegradability	An ideal option for tissue engineering applications, appropriate for mimicking tissue, vascular cell culture, nontoxicity and mechanical strength.

* METAC: 2-(methacryloyloxy)ethyltrimethlammonium chloride.

**Table 3:** Crosslinking methods to design hydrogels.

Methods	Ref.	Category	Advantages
1. Chemically crosslinked gels	[129,130]	Crosslinking by radical polymerization	Water-soluble polymers can be achieved with an initiator and catalyst. Such a system is very efficient, and at ambient temperature, gel forms quickly. Water solubility, short-chain and solubility activity.
		Crosslinking by chemical reaction of interdependent groups	A group of polymer chains can be connected with covalent linkages due to their interdependent reactivity.
		Crosslinking by high energy irradiation	-
	[131]	Crosslinking using enzymes	In an equilibrium state (more than 90% water content), gelatin is formed.
2. Physically crosslinked gels	[128]	Crosslinking ionically	Very effective on the self-healing properties of hydrogels.
	[126]	Crosslinking by crystallization	By the process of freeze-thawing, a very elastic gel is formed.
	[132]	Physically crosslinked hydrogels from by graft copolymers	The uniform structure is formed in water.
3. Crosslinking by hydrogen bonds	[133]	-	Swelling is a function of pH.
4. Crosslinking by protein interactions	[134]	Use of genetically designed proteins	By manipulating genetic DNA code, physical and chemical properties are controllable parameters (More related to Genetic Engineering).
		Crosslinking by antigen-antibody interactions	Good for drug delivery to target specific antigens.

**Table 4:** Effects of monomers and polymers materials on hydrogels’ CoF.

Author	Ref.	Year	Materials	CoF	Findings
Gong et al.	[142]	2001	PAMPS	0.001	Polymers with dangling chains reduce CoF substantially.
Covert et al.	[77]	2003	PVA-c	*Stc.: 0.285 Dyn.: 0.143	Friction significantly depends on material stiffness and toughness.
Yasuda et al.	[117]	2005	PAMPS	0.040	Excellent wear properties compared to UHMWPE.
Lin et al.	[181]	2009	PAAm-Alg-SNPs	0.00026	The incorporation of nano-silica significantly increased the compressive strength and fracture toughness but lowered the cross-linking density and CoF.
Arkaki et al.	[182]	2010	PAMPS/PDMAAm	0.029	Low CoF on normal cartilage, no significant detrimental effects on counterface cartilage.
Liao et al.	[161]	2013	PAAm-Alg-caprolactone	0.150	Tough material and potential for cell-based artificial cartilage.
Li et al.	[183]	2016	PVA on cartilage	0.114	The CoF significantly depends on load and speed.
Zhang et al.	[119]	2017	PAAm-AAc-METAC	<0.07	Salt leaching method was used to modulate porosity on the surface of the hydrogel, and it reduced CoF.
Arjmandi et al.	[26]	2018	PAAm-Alg	0.01	Less material was removed under higher sliding speed in their tribology tests.
Li et al.	[184]	2020	PAAm and different crosslinking concentrations	0.008–0.04	In the low normal force regime, friction is mainly adhesion-controlled and increases with polymer volume fraction. In the high normal force regime, friction is predominantly load-controlled and shows a slow increase with normal force.

*Stc: Static; *Dyn: Dynamics; *PAMPS: Poly 2-acrylamido−2-methyl−1-propanesulfonic acid; *PVA-c: Poly vinyl-alcohol cryogel; *PAMPS: Poly (2-acrylamide−2-metyl-propane sulfonic acid) and polyacrylamide; *PAMPS/PDMAAm: Poly-(2-Acrylamido−2-methylpropane sulfonic acid)/poly-(N,N’-dimetyl acrylamide).

## Data Availability

The data presented in this study are available on request from the corresponding author.
